# Immunogenicity, safety and clinical outcomes of the SARS-CoV-2 BNT162b2 vaccine in adolescents with type 1 diabetes

**DOI:** 10.3389/fped.2023.1191706

**Published:** 2023-06-26

**Authors:** Hamdi Cihan Emeksiz, Merve Nur Hepokur, Sibel Ergin Şahin, Banu Nursoy Şirvan, Burçin Çiçek, Aşan Önder, Metin Yıldız, Derya Karaman Aksakal, Aysun Bideci, Hüsnü Fahri Ovalı, Ferruh İşman

**Affiliations:** ^1^ Department of Pediatric Endocrinology, Professor Doctor Süleyman Yalçın City Hospital, Istanbul Medeniyet University, Istanbul, Türkiye; ^2^ Department of Pediatric Endocrinology, Gazi University Hospital, Ankara, Türkiye; ^3^ Department of Pediatrics, Professor Doctor Süleyman Yalçın City Hospital, Istanbul Medeniyet University, Istanbul, Türkiye; ^4^ Department of Biochemistry, Professor Doctor Süleyman Yalçın City Hospital, Istanbul Medeniyet University, Istanbul, Türkiye

**Keywords:** BNT162b2 vaccine, COVID-19, immunogenicity, type 1 diabetes, adolescents, SARS-CoV-2, breakthrough infection, safety

## Abstract

**Introduction:**

The mRNA-based BNT162b2 (Pfizer-BioNTech) vaccine has been shown to elicit robust systemic immune response and confer substantial protection against the severe coronavirus disease (COVID-19), with a favorable safety profile in adolescents. However, no data exist regarding immunogenicity, reactogenicity and clinical outcomes of COVID-19 vaccines in adolescents with type 1 diabetes (T1D). In this prospective observational cohort study, we examined the humoral immune responses and side effects induced by the BNT162b2 vaccine, as well as, the rate and symptomatology of laboratory-confirmed COVID-19 vaccine breakthrough infections after completion of dual-dose BNT162b2 vaccination in adolescents with T1D and compared their data with those of healthy control adolescents. The new data obtained after the vaccination of adolescents with T1D could guide their further COVID-19 vaccination schedule.

**Methods:**

A total of 132 adolescents with T1D and 71 controls were enrolled in the study, of whom 81 COVID-19 infection-naive adolescents with T1D (patient group) and 40 COVID-19 infection-naive controls (control group) were eligible for the final analysis. The response of participants to the BNT162b2 vaccine was assessed by measuring their serum IgG antibodies to the spike protein of severe acute respiratory syndrome coronavirus 2 (SARS-CoV-2), 4–6 weeks after the receipt of first and second vaccine doses. Data about the adverse events of the vaccine was collected after the receipt of each vaccine dose. The rate of COVID-19 vaccine breakthrough infections was evaluated in the 6-month period following second vaccination.

**Results:**

After vaccinations, adolescents with T1D and controls exhibited similar, highly robust increments in anti-SARS-CoV-2 IgG titers. All the participants in the patient and control groups developed anti-SARS-CoV-2 IgG titers over 1,050 AU/ml after the second vaccine dose which is associated with a neutralizing effect. None of the participants experienced severe adverse events. The rate of breakthrough infections in the patient group was similar to that in the control group. Clinical symptomatology was mild in all cases.

**Conclusion:**

Our findings suggest that two-dose BNT162b2 vaccine administered to adolescents with T1D elicits robust humoral immune response, with a favorable safety profile and can provide protection against severe SARS-CoV-2 infection similar to that in healthy adolescents.

## Introduction

The coronavirus disease first described in 2019 (COVID-19) is a communicable respiratory illness caused by a new strain of coronavirus called severe acute respiratory syndrome coronavirus 2 (SARS-CoV-2) (1). The virus has spread rapidly around the world and the extent of COVID-19 outbreak was declared a global pandemic in March 2020. It has devastating impact on public health, with COVID-19-related morbidity and mortality reaching significant figures. As of March 16, 2023, more than 760 million confirmed COVID-19 cases and more than 6.8 million deaths have been reported (2). Centers for disease control and prevention (CDC) identified certain risk factors for worsening SARS-CoV-2 infection (3). In addition to being unvaccinated, older age and certain chronic comorbidities are associated with more severe illness, higher hospitalization rates and mortality from COVID-19 (4). Children and adolescents appear to have milder form of COVID-19 but in rare cases they can develop critical acute disease, a severe post-infectious condition called multisystem inflammatory syndrome in children (MIS-C) or even long COVID-19 (5, 6). As in adults, chronic comorbidities such as type 1 diabetes (T1D), type 2 diabetes (T2D), asthma, chronic lung disease, cardiovascular disease, sickle cell disease and impaired immunity in children and adolescents can increase the risk for worse outcomes of COVID-19 (4, 7–14). Children and adolescents with T1D were found to have a 2.38 times greater risk of developing severe disease and a 4.60 times higher risk of hospitalization due to SARS-CoV-2 infection when compared to those without diabetes (13, 14). SARS-CoV-2 infection may induce metabolic derangements in children and adolescents particularly with poorly controlled T1D, which in turn may lead to diabetic ketoacidosis (DKA), clinical detoriation and finally increased rate of hospitalization. Therefore, taken together, vaccination against COVID-19 has been highly recommended for children and adolescents with T1D (15). On the other hand, since either form of diabetes is associated with persistent and profound impairments in both innate and acquired immunity (16, 17), whether the immune system of people with diabetes will be able to mount sufficient humoral immune response following COVID-19 vaccination has remained in question.

Some studies noted that adults with diabetes had reduced antibody response to hepatitis B vaccines, whereas less consistent results were reported for varicella-zoster and influenza vaccines (18). Likewise, certain pediatric studies found that serum antibody titers against hepatitis B surface antigen and pneumococcal antigens were lower in children and adolescents with T1D than in controls following hepatitis B and unconjugated pneumococcal polysaccharide vaccine administration (19–23). Therefore, concerns about the effectiveness of SARS-CoV-2 vaccines in people with diabetes have led to the investigation of their immunogenicity after vaccination against COVID-19. In the majority of studies conducted so far, lower humoral immune response and efficacy was reported in people with diabetes as compared to controls without diabetes (24–30). Most of that research, however, was carried out among adult patients with T2D. No data as yet exist on the use of SARS-CoV-2 vaccines in adolescents with T1D. In this context, we aimed to investigate the humoral immune response and side effect profile of the BNT162b2 vaccine, and the frequency and clinical outcomes of COVID-19 vaccine breakthrough infections in COVID-19 infection-naive adolescents with T1D in comparison to COVID-19 infection-naive healthy control adolescents.

## Methods

### Patients and study design

**Figure 1:**
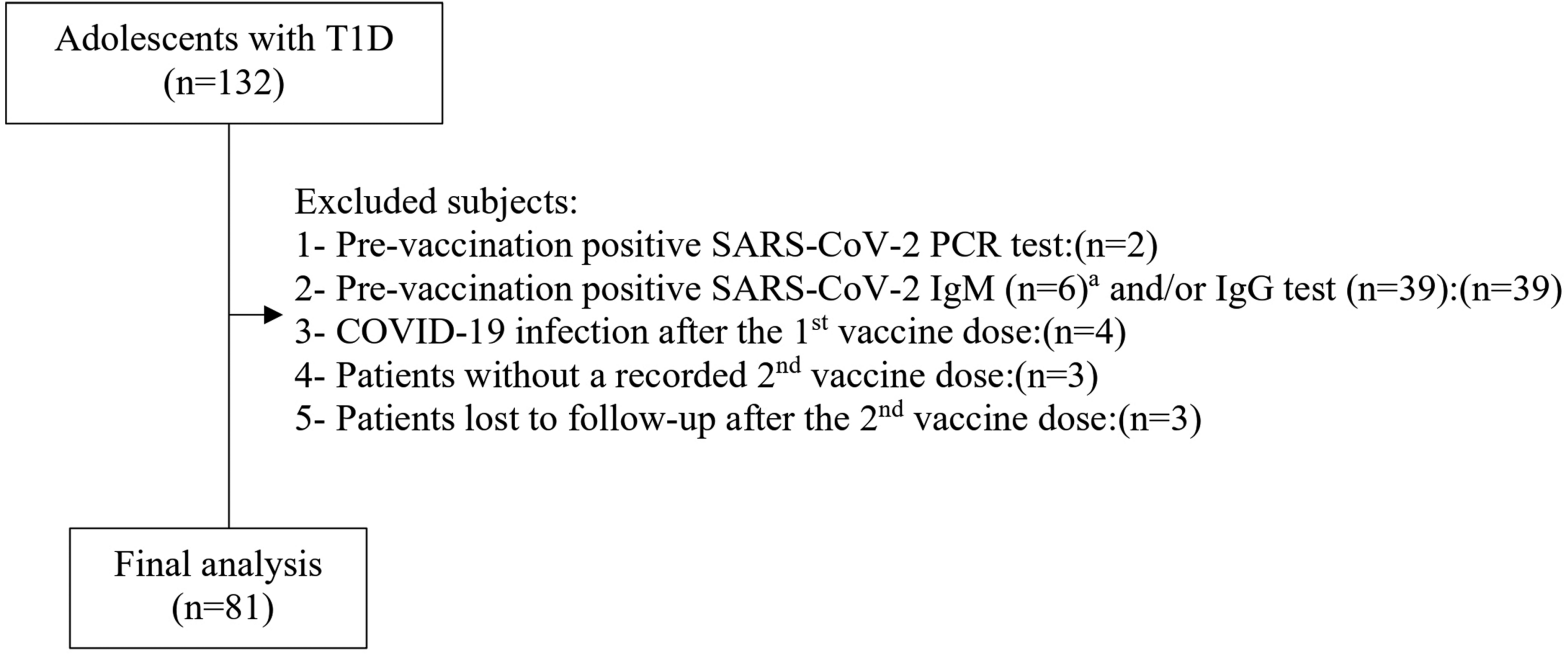
Patient group selection flow chart. ^a^All cases with a positive SARS-CoV-2 IgM test were also positive for SARS-CoV-2 IgG test, T1D., type 1 diabetes.

**Figure 2:**
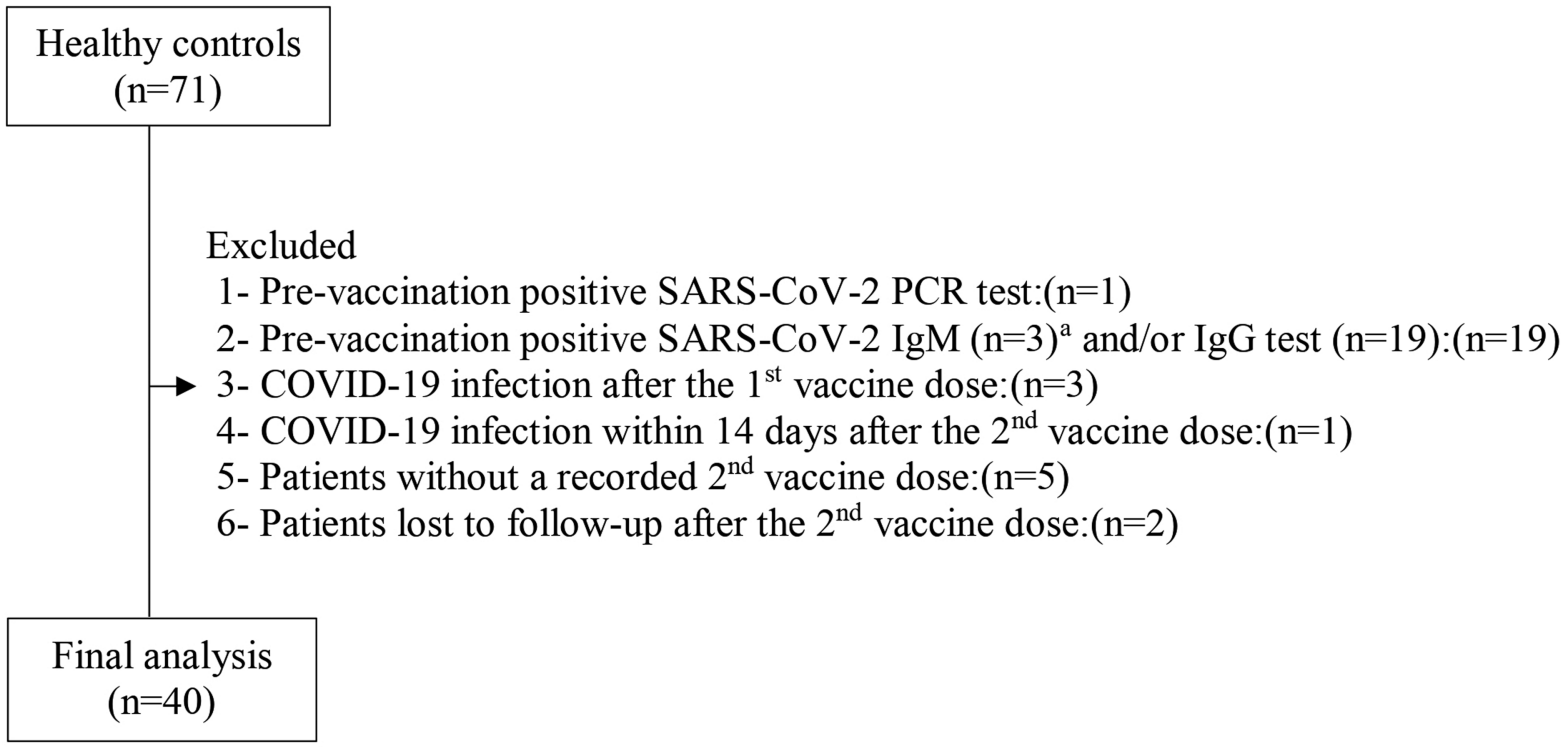
Control group selection flow chart. ^a^All cases with a positive SARS-CoV-2 IgM test were also positive for SARS-CoV-2 IgG test.

This prospective study was performed during the period from September 3, 2021, to August 1, 2022. It included 132 adolescents with T1D followed at Istanbul Medeniyet University, Professor Doctor Süleyman Yalçın City Hospital, Pediatric Endocrinology Clinic, Istanbul, Turkey, and 71 healthy control adolescents. They all were volunteers who arranged an appointment for BNT162b2 vaccination at Professor Doctor Süleyman Yalçın City Hospital which was designated as the pandemic hospital in the Kadıköy district of Istanbul by the Turkish Ministry of Health. Diagnosis and classification of T1D was made according to American Diabetes Association criteria (31). Patients with no history of chronic disease other than T1D, no previous episode of COVID-19, and no respiratory symptoms up to 14 days before the study and those who were not receiving systematic treatment with corticosteroids and/or immunosuppressant medications were included in the study. Whereas, the inclusion criteria of the healthy controls were good health, not receiving systematic treatment with corticosteroids and/or immunosuppressant medications, and having no history of chronic disease, no previous episode of COVID-19, and no respiratory symptoms up to 14 days before the study. None of the subjects had previously been vaccinated against COVID-19. Patients (*n* = 132) and controls (*n* = 71) who fulfilled the preliminary inclusion criteria were subjected to the SARS-CoV-2 real time reverse transcriptase polymerase chain reaction test (RT-PCR) 2 days before the administration of the first BNT162b2 vaccine dose for the identification of asymptomatic SARS-CoV-2 infection. Those with a positive SARS-CoV-2 RT-PCR test result were also precluded. Within the 2 days following RT-PCR testing, just before the first dose of vaccine was administered, blood samples were obtained from participants with a negative SARS-CoV-2 RT-PCR test result to measure anti-SARS-CoV-2 spike IgM and IgG antibodies to exclude prior SARS-CoV-2 infection. Finally, 81 COVID-19 infection-naive adolescents with T1D and 40 COVID-19 infection-naive healthy controls were included (Figures 1, 2). None of these participants had any clinical evidence of COVID-19 infection during the period between first and second vaccination. Their clinical condition and infection-naive status within that time span was checked by phone call and by reviewing electronic medical records based on the results of SARS-CoV-2 RT-PCR tests. After completion of the blood sample collections for the measurement of anti-SARS-CoV-2 spike IgG titers, the participants remained under 6-month follow-up to find out the rate of COVID-19 vaccine breakthrough infections after the second vaccination. Occurrence of COVID-19 infection ≥14 days after administration of two doses of BNT162b2 vaccine was defined as COVID-19 vaccine breakthrough infection (32).

Data for adolescents with T1D were collected from the medical records. Their recent mean HbA1c level was calculated from the HbA1c levels in the last 2 years. HbA1c ≥ 7% (53 mmol/mol) represents moderate-poor glycemic control (33). All participants underwent physical examination. *Z*-scores for weight, height, and body mass index (BMI) were calculated using the reference values for Turkish children.

At the time of data collection, the only vaccine approved by the Turkish Ministry of Health (TMH) for the vaccination of adolescents was the Pfizer-BioNTech (BNT162b2) vaccine. According to the TMH's recommendation, the vaccine was administered to all participants as two shots and given a minimum of 28 days apart. In Turkey, children ≥12 years of age with chronic diseases and healthy ones ≥12 years of age started to be vaccinated as of August 16, 2021, and as of September 3, 2021, respectively. Data on the adverse events of the BNT162b2 vaccine including severe allergic reaction, local injection site reaction (pain, swelling, redness), systemic reaction (elevated body temperature >38°C, headache, fatigue, myalgia, arthralgia, nausea, diarrhea, chills) or hospitalization, were collected by phone call a week and 2 weeks after the receipt of each vaccine dose.

Blood samples were taken from all participants with a negative pre-vaccination SARS-CoV-2 RT-PCR test result for the measurement of anti-SARS-CoV-2 spike IgM and IgG antibodies. Anti-SARS-CoV-2 IgM antibodies were measured just before the first vaccine dose (T0), while anti-SARS-CoV-2 IgG antibodies were measured just before the first vaccine dose (T0), a minimum of 28 days after the first vaccine dose (T1), and a minimum of 28 days after the second vaccine dose (T2). The blood samples were centrifuged 10 min after collection at 400× *g* for 10 min at room temperature to isolate serum. Serum samples were then aliquoted and stored −80°C until all assays were performed.

### SARS-CoV-2 spike IgM and IgG antibodies detection

For the measurement of anti-SARS-CoV-2 spike IgM antibodies, the AdviseDx SARS-CoV-2 IgM assay was used. It has been granted Emergency Use Authorization by the US Food and Drug administration. IgM testing was performed on the Abbott Architect platform per manufacturer instructions. The test is a chemiluminescent microparticle (CMIA) assay for qualitative assessment of IgM antibodies to the spike protein of SARS-CoV-2 in human serum and plasma sample. A vendor recommended cut-off of 1.0 (index value) for reactivity/positivity of infection was applied (34).

For the measurement of anti-SARS-CoV-2 spike IgG antibodies, SARS-CoV-2 IgG II quantitative testing was performed on the Abbott Architect platform in accordance with manufacturer's package insert. In this antibody CMIA test, the SARS-CoV-2 antigen coated paramagnetic microparticles bind to the IgG antibodies that attach to the virus’ spike protein in human serum and plasma sample. The resulting chemiluminescence in relative light units (RLU) following the addition of anti-human IgG-labeled in comparison with the IgG II calibrator/standard indicates the strength of response, which reflects the quantity of IgG present. The titers ≥50 AU/ml in this test are considered as positive. This quantitative measurement of IgG was reported to be 100% compatible with the plaque reduction neutralization test (PRNT), and a concentration of 1,050 AU/ml was associated with a 1:80 dilution of PRNT (35).

## Statistical analyses

The IBM SPSS statistic 26 package program was used to evaluate the data. Qualitative data are presented as number and percentage, and quantitative data are presented as mean (standard deviation) and median (interquartile range 25–75). The Shapiro-Wilk test was used for test of normality. The chi- square and the Fisher's exact test were used in the evaluation of qualitative data whereas the Student's *t* test and the Mann Whitney *U* test were used in the comparison of quantitative data. The Spearman analysis was used for the correlation analysis and *P* < 0.05 value was considered significant in all analysis.

## Results

### Characteristics of patients and controls

Of the 132 adolescents with T1D and the 71 healthy control adolescents, 51 and 31 participants were excluded, respectively (reasons shown in the flowcharts of Figures 1, 2). A total of 81 COVID-19 infection-naive adolescents with T1D (patient group) and 40 COVID-19 infection-naive healthy controls (control group) met the inclusion criteria for the final analysis. None of these participants had any clinical evidence of COVID-19 breakthrough infection during the period between the first and second vaccination.

No significant differences were found in demographic characteristics between the groups (*P* > 0.05 for all) (Table 1). All participants were aged 12< and <18 years. In the patient group, 72 participants (90%) had moderately/poorly controlled T1D [HbA1c ≥ 7% (53 mmol/mol)].

**Table 1:** Characteristics and anti-SARS-CoV-2 IgG antibody response of the study participants.

Variables	COVID-19 infection-naive adolescents with T1D (*n* = 81)	COVID-19 infection-naive healthy controls (*n* = 40)	*P* value
Age (years) mean ± standard deviation	14.68 ± 0.22	14.75 ± 0.26	0.987
Male/Female, *n* (%)	42 (51.8)/39 (48.2)	16 (40.0)/24 (60.0)	0.220
Height *z*-score mean ± standard deviation	0.10 ± 1.0	0.11 ± 1.44	0.826
Body mass index *z*-score median (25th, 75th percentiles)	−0.33 (−0.74, 0.39)	0.32 (−0.51, 0.77)	0.063
Diabetes duration (years) median (25th, 75th percentiles)	4.3 (2.3, 7.3)	−	−
HbA1c^a^ median (25th, 75th percentiles)	8.6 [7.6, 10.3]	−	−
Microvascular complications Microalbuminuria, *n* (%)	2 (2.46%)	−	−
T1 (days) median (25th, 75th percentiles)	28 (28, 28)	28 (28, 31)	0.060
T2 (days) median (25th, 75th percentiles)	31 (30, 32)	31 (30, 33.75)	0.187
Anti-SARS-CoV-2 IgM index at T0^b^ median (25th, 75th percentiles)	0.05 (0.04, 0.07)	0.05 (0.04, 0.09)	0.381
Anti-SARS-CoV-2 IgG titer in AU/ml at T0^c^ median (25th, 75th percentiles)	2.30 (1.35, 3.65)	2.65 (1.52, 3.68)	0.656
Anti-SARS-CoV-2 IgG titer ≥50 AU/ml at T1, *n* (%)	81 (100)	40 (100)	-
Anti-SARS-CoV-2 IgG titer ≥1,050 AU/ml at T1, *n* (%)	58 (71.6)	32 (80)	0.322
Anti-SARS-CoV-2 IgG titer in AU/ml at T1 median (25th, 75th percentiles)	1,558.70 (939.95, 2,692.60)	2,167.75 (1,205.33, 3,724.80)	0.079
Anti-SARS-CoV-2 IgG titer ≥1,050 AU/ml at T2, *n* (%)	81 (100)	40 (100)	−
Anti-SARS-CoV-2 IgG titer in AU/ml at T2 median (25th, 75th percentiles)	18,606.20 (12,204.20, 31,104.65)	22,647.60 (16,351.73, 38,943.63)	0.042

^a^ Mean HbA1c level in the last 2 years.

^b^ Anti-SARS-CoV-2 IgM index <1 indicates negativity.

^c^ Anti-SARS-CoV-2 IgG titer <50 AU/ml indicates negativity.

T0, anti-SARS-CoV-2 IgM and IgG measurements just before the first vaccine dose; T1, days from first vaccine dose to anti-SARS-CoV-2 IgG measurement; T2, days from second vaccine dose to anti-SARS-CoV-2 IgG measurement; HbA1c, glycosylated hemoglobin; T1D, type 1 diabetes; AU, arbitrary unit.

### SARS-CoV-2 RT-PCR test

Scheduled SARS-CoV-2 RT-PCR testing was performed in two time periods to rule out asymptomatic SARS-CoV-2 infection. Firstly, all patients (*n* = 132) and controls (*n* = 71) who fulfilled the preliminary inclusion criteria were subjected to SARS-CoV-2 RT-PCR test 2 days before the first vaccination. Amongst, 2 patients and 1 control who had a positive pre-vaccination test result were excluded from the study (Figures 1, 2). Secondly, between the first and second vaccination, weekly SARS-CoV-2 RT-PCR testing for 4 weeks was scheduled for all participants. However, there was no full participation in the SARS-CoV-2 RT-PCR testing in the first 3 weeks. The fourth-week SARS-CoV-2 RT-PCR test was performed just before the second vaccination, therefore it could be sent on all participants. At the first, second, third and fourth weeks after the first vaccination, 29 patients and 18 controls, 22 patients and 13 controls, 15 patients and 7 controls and 82 patients and 41 controls underwent scheduled SARS-CoV-2 RT-PCR testing, respectively. At the second week, 2 patients, and at the fourth week, 1 patient had a positive SARS-CoV-2 RT-PCR test result whereas at the first, second and fourth weeks, 1 control for each week had a positive SARS-CoV-2 RT-PCR test result. The participants without a SARS-COV-2 RT-PCR test at the first, second and third week were tracked by phone. Amongst, those with suspected COVID-19 symptomatology and/or a history of close contact agreed to provide samples for SARS-CoV-2 RT-PCR test. By this way, the SARS-CoV-2 RT-PCR test was performed in an additional 3 patients and 1 control, 2 patients and 2 controls, and 1 patient after the first, second and third weeks, respectively. Among these, one patient with clinical symptoms had a positive SARS-CoV-2 RT-PCR test result. In total, 4 patients and 3 controls with a positive test result during the time span between the first and second vaccinations were excluded from the study (Figures 1, 2). All were symptomatic.

### Immunogenicity

**Figure 3:**
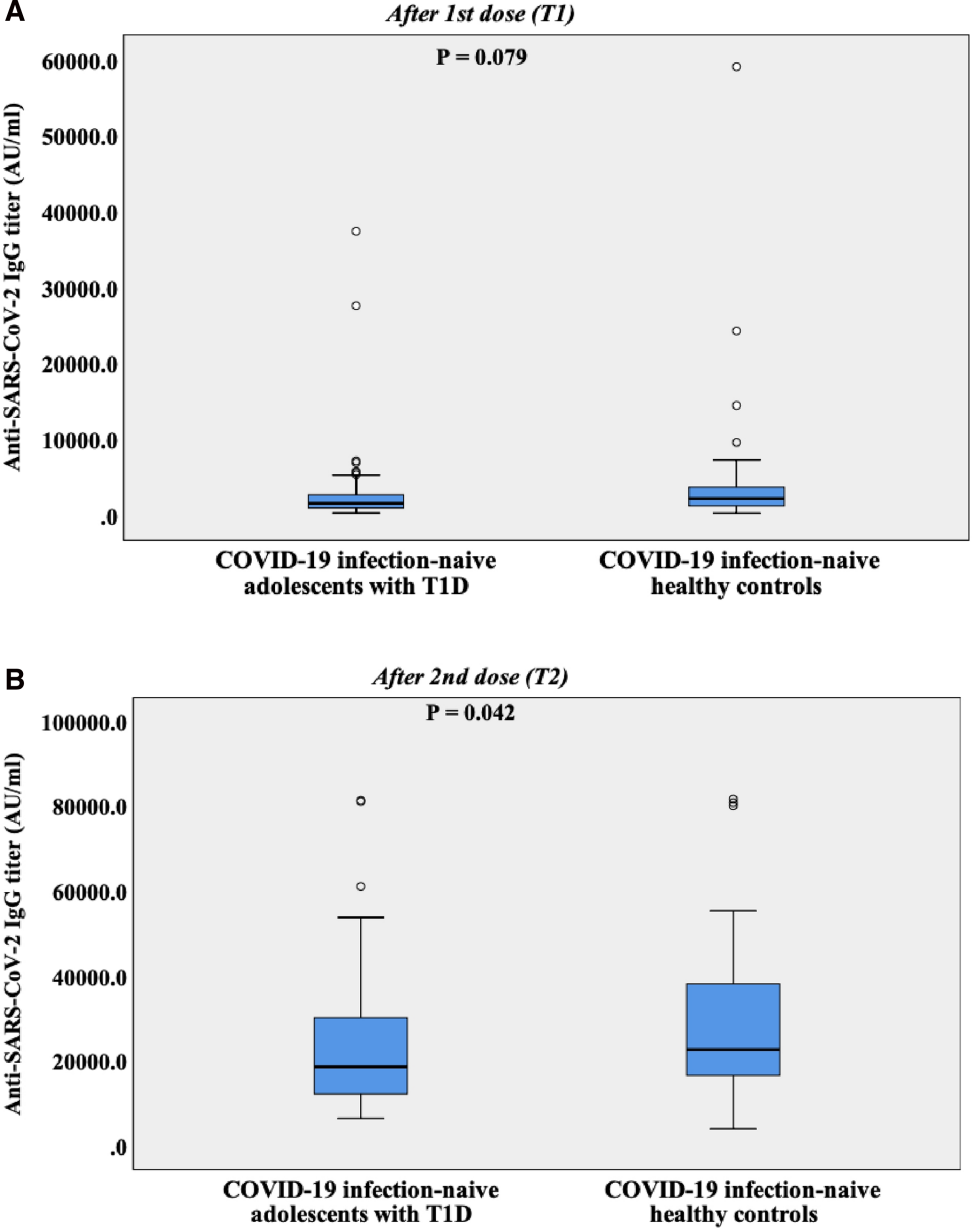
(**A**) Box plot representing anti-SARS-CoV-2 spike IgG levels of COVID-19 infection-naive adolescents with T1D and COVID-19 infection-naive healthy controls after the first vaccine dose. Median days from first dose to anti-SARS-CoV-2 spike IgG measurement (T1) was 28 days. AU, arbitrary unit; T1D, type 1 diabetes. (**B**) Box plot representing anti-SARS-CoV-2 spike IgG levels of COVID-19 infection-naive adolescents with T1D and COVID-19 infection-naive healthy controls after the second vaccine dose. Median days from second dose to anti-SARS-CoV-2 spike IgG measurement (T2) was 31 days. AU, arbitrary unit; T1D, type 1 diabetes.

There were no differences in T1 (median 28 days) and T2 (median 28 days) between the groups (*P* = 0.060, *P* = 0.187, respectively) (Table 1). At T1, anti-SARS-CoV-2 IgG ≥ 50 AU/ml were detected in all adolescents with T1D and in all healthy controls. The median anti-SARS-CoV-2 IgG titer was 1,558.70 AU/ml in the patient group and 2,167.75 AU/ml in the control group. The difference between the two groups was not statistically significant (*P* = 0.079). Anti-SARS-CoV-2 IgG titers > 1,050 AU/ml were detected in 71.6% of the patient group, and in 80% of the control group; the difference was statistically insignificant (*P* = 0.322) (Table 1, Figure 3).

At T2, the median anti-SARS-CoV-2 IgG titer increased to 18,606.20 AU/ml in the patient group and to 22,647.60 AU/ml in the control group. The difference between the two groups was slightly significant (*P* = 0.042). All patients and controls mounted anti-SARS-CoV-2 IgG titers >1,050 AU/ml at T2, which is associated with a neutralizing effect (Table 1, Figure 3).

In the total sample of patients and controls, no significant gender differences were found in the anti-SARS-CoV-2 IgG titers at either T1 or T2. Similarly, gender differences were not detected in the patient group for anti-SARS-CoV-2 IgG titers at the same time points.

Of the 132 adolescents with T1D and the 71 healthy controls, 39 (29.5%) and 19 (26.8%) participants with a negative pre-vaccination SARS-CoV-2 RT-PCR test were excluded respectively, because of their pre-vaccination positive SARS-CoV-2 IgM and/or IgG tests results (Figures 1, 2). When these subjects were added to the COVID-19 infection-naive patient and infection-naive control groups respectively, and these groups were compared for anti-SARS-CoV-2 IgG titers, no significant differences were found between the two groups at T1 and T2 (Table 2).

**Table 2:** Comparison of anti-SARS-CoV-2 IgG antibody responses between adolescents with T1D and healthy controls regardless of pre-vaccination SARS-CoV-2 IgM and IgG test results

Variables	Adolescents with T1D [*n* = 120 (81 + 39)]	Healthy controls [*n* = 59 (40 + 19)]	*P* value
T1 (days) median (25th, 75th percentiles)	28 (28, 31)	28 (28, 30)	0.144
T2 (days) median (25th, 75th percentiles)	31 (29, 33)	31 (29, 31)	0.342
Anti-SARS-CoV-2 IgG titer ≥50 AU/ml at T1, *n* (%)	120 (100%)	59 (100%)	−
Anti-SARS-CoV-2 IgG titer ≥1,050 AU/ml at T1, *n* (%)	98 (81.7%)	51 (86.4%)	0.421
Anti-SARS-CoV-2 IgG titer in AU/ml at T1 median (25th, 75th percentiles)	2,460.55 (1,318.53, 25,385.8)	3,603.60 (1,538.80, 24,276.70)	0.260
Anti-SARS-CoV-2 IgG titer ≥1,050 AU/ml at T2, *n* (%)	120 (100%)	59 (100%)	−
Anti-SARS-CoV-2 IgG titer in AU/ml at T2 median (25th, 75th percentiles)	22,695.4 (13,565.87, 36,815.85)	25,366.0 (17,253.1, 42,008.0)	0.193

T1, days from first vaccine dose to anti-SARS-CoV-2 IgG measurement; T2, days from second vaccine dose to anti-SARS-CoV-2 IgG measurement, T1D, type 1 diabetes, AU, arbitrary unit.

Weak negative correlations were found between anti-SARS-CoV-2 IgG titers at both T1 and T2 and diabetes duration (*r* = −0.237, *P* = 0.034; *r* = −0.247, *P* = 0.026, respectively). Except for these, there were no associations between the parameters evaluated in this study (*P >* 0.05 for all).

### Safety

After the second vaccination, increments in local and systemic side effects were observed in both groups compared to the first vaccination. No significant differences were found between adolescents with T1D and healthy controls with regard to local and systemic side effects after the first and second vaccine doses (*P >* 0.05 for all). The most common local and systemic side effect were local pain and fatique, respectively (Supplementary Figure S1). None of the participants experienced severe adverse events. All side effects were observed within 72 hours after both first and second vaccinations.

### Clinical outcomes

In the 6-month period after the second vaccination, rates and clinical features of RT-PCR-confirmed COVID-19 vaccine breakthrough infections were analyzed amongst the 81 COVID-19 infection-naive adolescents with T1D and 40 COVID-19 infection-naive healthy controls. SARS-CoV-2 RT-PCR test was performed on those who had suspected COVID-19 symptom(s) or had a history of close contact with a SARS-CoV-2 positive person. Patients contacted us by phone in case these risk factors for COVID-19 emerged. Of the 81 infection-naive adolescents with T1D and of the 40 infection-naive healthy controls, 18 patients (22.2%) and 7 controls (17.5%) who received the third dose of BNT162b2 vaccine (booster dose) in the above-mentioned time span, were excluded. Finally, of the 63 adolescents with T1D and of the 33 controls, 9 patients (14.3%) and 8 controls (24.2%) had vaccine breakthrough infections, respectively. All of them had clinical symptoms. None of them received the diagnosis of COVID-19 within 21 days after the second vaccination. The rate of symptomatic vaccine breakthrough infections did not differ between the patient and control groups (*P* = 0.225). The median interval between the receipt of the second dose of vaccine and vaccine breakthrough infection diagnosis was 110 days (range, 101–153) and 71 days (range, 37–144) in the patient and control groups, respectively. None of the cases in the patient group developed COVID-19 breakthrough infection within 3 months after the second vaccination. Clinical symptoms of the vaccine breakthrough infections was mild in all cases. Most common symptom of breakthrough infection was fever, followed by malaise and headache (Supplementary Figure S2). Only one male adolescent with poorly controlled T1D, on insulin pump treatment, was hospitalized due to COVID-19 induced severe DKA and discharged 3 days later.

## Discussion

To our knowledge, this is the first study to evaluate the immunogenicity, side effect profile and clinical outcomes of COVID-19 vaccination in adolescents with T1D. The results of this prospective observational cohort study showed that infection-naive adolescents with T1D display a robust humoral immune response to BNT162b2 vaccine, measured according to anti-SARS-CoV-2 spike IgG, that is comparable to that of infection-naive healthy controls. Regarding the adverse events after the uptake of the BNT162b2 vaccine, no significant differences were observed in local and systemic side effects between adolescents with T1D and healthy controls. No severe advers events were reported. In the 6-month follow-up period after the second vaccination, RT-PCR-confirmed vaccine breakthrough infections' incidence, symptomatology and clinical outcomes were similar between adolescents with T1D and healthy controls.

Impairments in the immune system of people with diabetes increase their susceptibility to infections and their risk of developing a lower antibody response to certain vaccines, such as hepatitis B and unconjugated pneumococcal polysaccharide vaccines (19–23). However, humoral immune response to SARS-CoV-2 vaccines in people with diabetes has been poorly studied. Most of the available data are derived from studies carried out mainly in adults with T2D (24–27). But the presence of certain heterogeneity among those studies such as study design features, age range of participants, type of vaccines administered, kits used for antibody testing, antibody measurement methods, COVID-19-naive status of participants, diabetic participants' glycemic control and time from first and second vaccination to antibody testing might limit the strength of conclusions made based upon their data. Despite those differences, two recent systematic reviews by Soetedjo et al. and Boroumand et al. reported that adult patients with T2D had lower antibody responses and seroconversion rates than controls and Soetedjo et al. suggested that this specific population should be prioritized for booster doses (36, 37).

Regarding the humoral immune response of adults with T1D, two studies thus far have extensively evaluated the impact of T1D on post-vaccination anti-SARS-CoV-2 antibody responses, however, with contradictory results (28, 29). In one of these studies, D'Addio et al. reported that both infection-naive patients with T1D and infection-naive healthy controls without diabetes exhibited a similar increase in anti-SARS-CoV-2 spike IgG titers 1 month after their first and second vaccine doses (28). Contrary to this finding, a more recent study by D'Onofrio et al. noted that anti-SARS-CoV-2 spike IgG titers measured 1 month after the first BNT162b2 dose administration were significantly lower in patients with T1D compared to controls without diabetes (29). However, a common limitation in both studies was the age discrepancy between the study and control groups. Given that mRNA-based COVID-19 vaccines induce weaker antibody responses in older adults (38), significantly advanced age of the controls compared to the diabetic patients in the study by D'Addio et al. might have impacted the anti-SARS-CoV-2 IgG results, as well as their interpretation. On the other hand, in the study by D'Onofrio et al., advanced age of the patient group compared to the control group might have contributed to the higher anti-SARS-CoV-2 spike IgG titers measured in the latter group. Thus, strength of humoral immune response after COVID-19 vaccination in adult patients with T1D still remains obscure and further large-scale prospective research particularly with age-matched controls is warranted.

In our study, antibody responses to BNT162b2 vaccine were found very robust in both infection-naive adolescents with T1D and infection-naive healthy controls and they were similar between the two groups after both the first and second vaccine doses. Anti-SARS-CoV-2 IgG titers were positive (≥50 AU/ml) after the first dose and exceeded the threshold value associated with neutralizing effect (>1,050 AU/ml) after the second dose in all participants. As is known, children and adolescents display more robust humoral immune response to mRNA-based COVID-19 vaccines compared to adults (39, 40). Accordingly, as we compared our study with the adult study by D'Onofrio et al., where the brand of anti-SARS-CoV-2 IgG quantitative assay and COVID-19 vaccine used were the same, we observed that the anti-SARS-CoV-2 IgG titers of the T1D and control groups 1 month after the first BNT162b2 vaccination in our study were much higher than those of the T1D and control groups measured at the same time span in that study. After the second vaccination, anti-SARS-CoV-2 IgG titers of the infection-naive adolescents with T1D was slightly lower than that of the infection-naive healthy controls. Thus far, certain studies suggested that older age, chronic kidney disease, hypertension and overt microvascular complications were found to be associated with lower COVID-19 vaccine response in patients with diabetes (26, 29, 41). However, none of our patients had these risk factors. As known, patients with T1D show persistent and profound limitations in both innate and adaptive immunity, therefore vaccines may evoke a less efficient immune response in patients with diabetes (23). Accordingly, immune dysfunction already present in T1D might have contributed to this slightly significant difference in anti-SARS-CoV-2 IgG titers between adolescents with T1D and healthy controls after the second vaccination. On the other hand, comparison of anti-SARS-CoV-2 IgG titers between RT-PCR-negative adolescents with T1D and RT-PCR-negative healthy controls, regardless of SARS-CoV-2 IgM and IgG test results before the first vaccination, showed no significant difference in anti-SARS-CoV-2 IgG titers between the two groups after the second vaccination. In the light of all the aforementioned data, it could be suggested that T1D does not seem to have a remarkable impact on humoral immune response of adolescents with T1D against SARS-CoV-2, in the first decade of diabetes onset.

Several studies reported that the BNT162b2 vaccine has an acceptable safety profile in children and adolescents (39–44). Accordingly, none of the adolescents in our study experienced severe adverse events. Side effects of the BNT162b2 vaccine in pediatric populations are reported to be more frequent after the second vaccine dose (44). Likewise, local and systemic side effects increased after the second vaccination in both groups compared to the first vaccination, and their rates did not differ between the groups after the receipt of each vaccine dose. In line with the literature (44), the most common local and systemic side effects were local pain and fatique, respectively, reported similar for both groups after each vaccination. These data indicate that the side effect profile of the BNT162b2 vaccine in adolescents with T1D were not different from those reported in their peers so far. Thus, the BNT162b2 vaccine appears to be safe in adolescents with T1D.

The question of whether poor glycemic control is associated with decreased antibody response to SARS-CoV-2 vaccines in adult patients with diabetes was investigated in a few studies to date. In the CAVEAT study, T2D patients with HbA1c > 7% had significantly reduced neutralizing antibody levels 21 days after the second SARS-CoV-2 vaccine dose, compared to normoglycemic subjects and T2D patients with good glycemic control (HbA1c < 7%) (27). However, the association between HbA1c levels and anti-SARS-CoV-2 antibody titers was found weak in patients with T2D. In another study, D'Onofrio et al. found no association between antibody levels and, HbA1c and fasting blood glucose levels in both patients with T1D and T2D (29). The authors suggested that the relatively good glycemic control at baseline for both patients with T1D and T2D may have contributed to this neutral finding. In line with the former data, we did not find a correlation between HbA1c levels and anti-SARS-CoV-2 IgG titers in adolescents with T1D. However, poor/moderate glycemic control among most of our patients might have contributed to the lack of an association between these parameters. Further studies comparing anti-SARS-CoV-2 IgG titers between adolescents with good, moderate, and poorly controlled T1D and with similar diabetes duration are needed to clarify the effect of glycemic control on anti-SARS-CoV-2 antibody response in adolescents with T1D.

The inconsistency in methodology, especially the definition of COVID-19 vaccine breakthrough infections among different studies limits the comparison of findings. Nonetheless, several studies demonstrated that the two-dose BNT162b2 vaccine provides strong protection against SARS-CoV-2 delta variant in adolescents (40, 45, 46). However, as more transmissible novel strain, omicron variant became prevalent worldwide since the start of 2022, the vaccine effectiveness was found to be decreased remarkably (46–48). In this research, the 6-month post-vaccination follow-up period through which the cases with breakthrough infections were diagnosed coincided with the time span when the omicron variant displaced the previously dominant delta variant in Turkey (49, 50). At the end of the follow-up, no significant difference was found in the rate of symptomatic breakthrough infections between adolescents with T1D and healthy controls. None of the symptomatic breakthrough infections in both groups progressed to severe, critical, or fatal COVID-19. Taken together, these finding suggest that the pre-existing T1D in adolescents does not seem to convey an additional risk for vaccine breakthrough infections during the omicron surge. Thus, two-dose BNT162b2 vaccine provides substantial protection against severe COVID-19 in adolescents with T1D.

Although not significant, the rate of symptomatic breakthrough infection in the patient group was lower than that in the control group, and none of the adolescents with T1D had symptomatic breakthrough infection earlier than 3 months after the receipt of the second vaccine dose. The probability of rigorous implementation of COVID-19 control measures such as hand washing, mask wearing and physical distancing by adolescents with T1D and family members because of the common fear of hospitalization of the patient due to severe infection and/or infection induced DKA may have contributed to these outcomes.

This study has some limitations, including a small sample size, sole use of the BNT162b2 vaccine, measurement of anti-SARS-CoV-2 IgG titers after a relatively short period of time, lack of cellular immunity data and SARS-COV-2 variant analysis. Additionally, the SARS-CoV-2 RT-PCR test could not be sent on all participants to identify asymptomatic SARS-CoV-2 infection in the first 3 weeks after the first vaccination. Shortly after the onset of this study, the resumption of full-time, face-to-face elementary and midschool first-term education, which had been disrupted by pandemics for 1.5 years in Turkey and therefore the common desire of the parents for their children not to be absent from school, as well as the reluctance of some participants to provide frequent tests since taking samples from the oropharynx and nasopharynx for RT-PCR test being more irritating and uncomfortable for children compared to adults may have contributed to this outcome during the above-mentioned period. However, during the enrollment process of the participants, detection of positive SARS-COV-2 RT-PCR test result in only 2 of 132 patients and 1 of 71 controls before the first vaccination and presence of symptoms in all 4 patients and 3 controls with positive SARS-COV-2 RT-PCR test result between the first and second vaccination strengthens the possibility that the number of participants with asymptomatic SARS-COV-2 infection would be quite low between the first and second vaccination. Another limitation of this study was that the effect of no vaccination could not been evaluated in adolescents with T1D since most of the patients followed by our clinic voluntarily got vaccinated with the two doses of BNT162b2 vaccine. Lastly, growth curves of Turkish adolescents have higher values for height and BMI as compared to world health organization (WHO) standards, however, we do not think this difference impact the results, since only two participants in our study were found obese with respect to WHO growth charts.

## Conclusions

Two-dose Pfizer-BioNTech BNT162b2 vaccine elicited very robust humoral immune response, with a favorable safety profile in adolescents with T1D. During the omicron surge, the detection of similar breakthrough infection rates between patient and control groups and absence of severe breakthrough infections in both groups through 6 months of post-vaccination follow-up suggests that the two-dose BNT162b2 vaccine series administered to healthy adolescents can provide similar protection in adolescents with T1D. Further prospective, large scale and longer term studies are required to monitor the COVID-19 vaccine effectiveness in adolescents with T1D to guide their subsequent vaccination strategies with regard to changing dynamics of SARS-CoV-2 variants and waning humoral immunity.

## Data Availability

The raw data supporting the conclusions of this article will be made available by the authors, without undue reservation.
