# Supplementary material for: Immunogenicity, safety and clinical outcomes of the SARS-CoV-2 BNT162b2 vaccine in adolescents with type 1 diabetes
Source: Front Pediatr. 2023 Jun 26;11:1191706. doi: 10.3389/fped.2023.1191706 (PMC10331611; doi:10.3389/fped.2023.1191706)

Supplementary Figure 1A

Bar graph representing percentage of COVID-19 infection-naive adolescents with T1D and COVID-19 infection-naive healthy controls reporting local and systemic reactions after dose 1 of the BNT162b2 vaccine.

Abbreviations: T1D, type 1 diabetes.

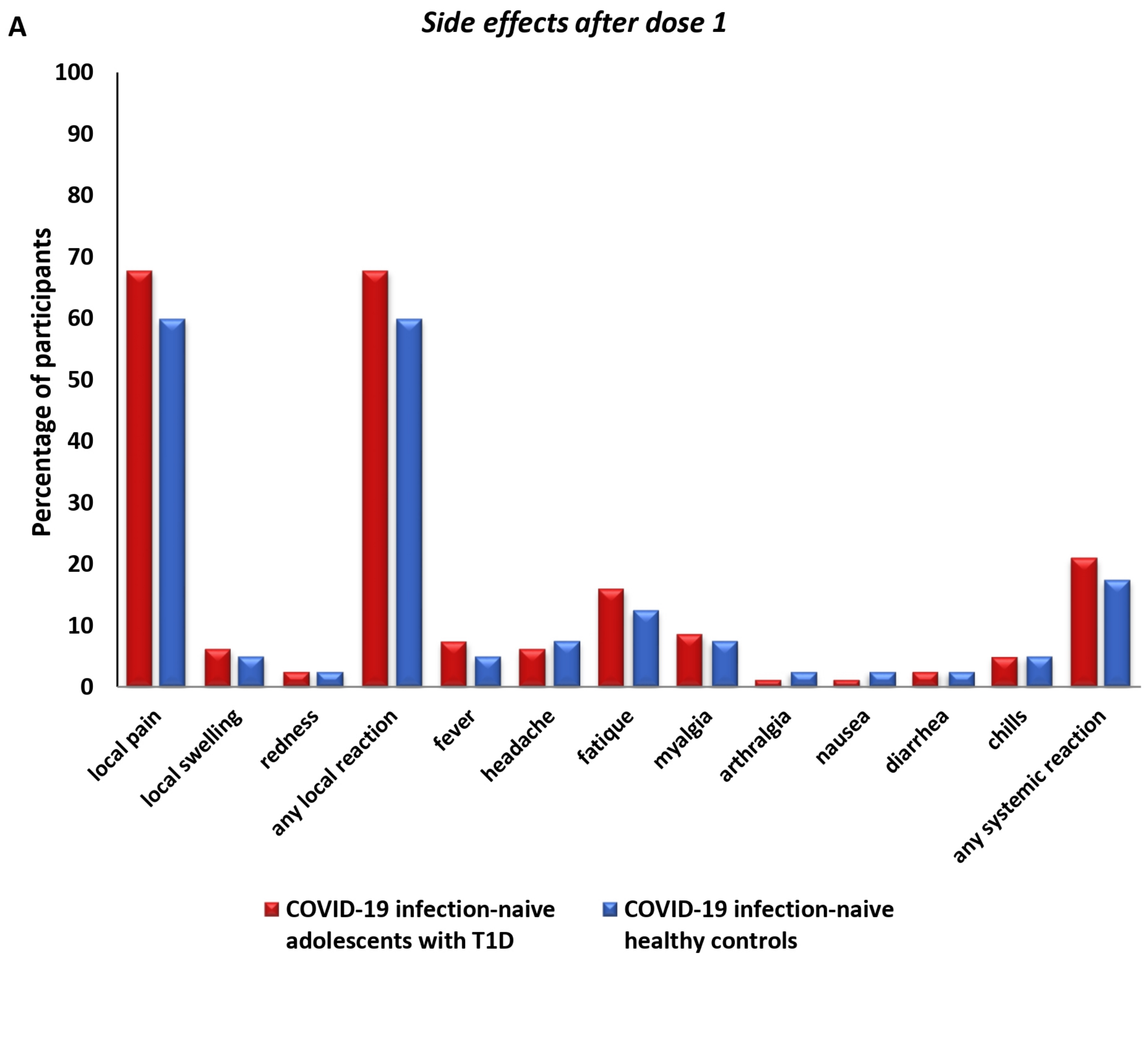

Supplementary Figure 1B

Bar graph representing percentage of COVID-19 infection-naive adolescents with T1D and COVID-19 infection-naive healthy controls reporting local and systemic reactions after dose 2 of the BNT162b2 vaccine.

Abbreviations: T1D, type 1 diabetes.

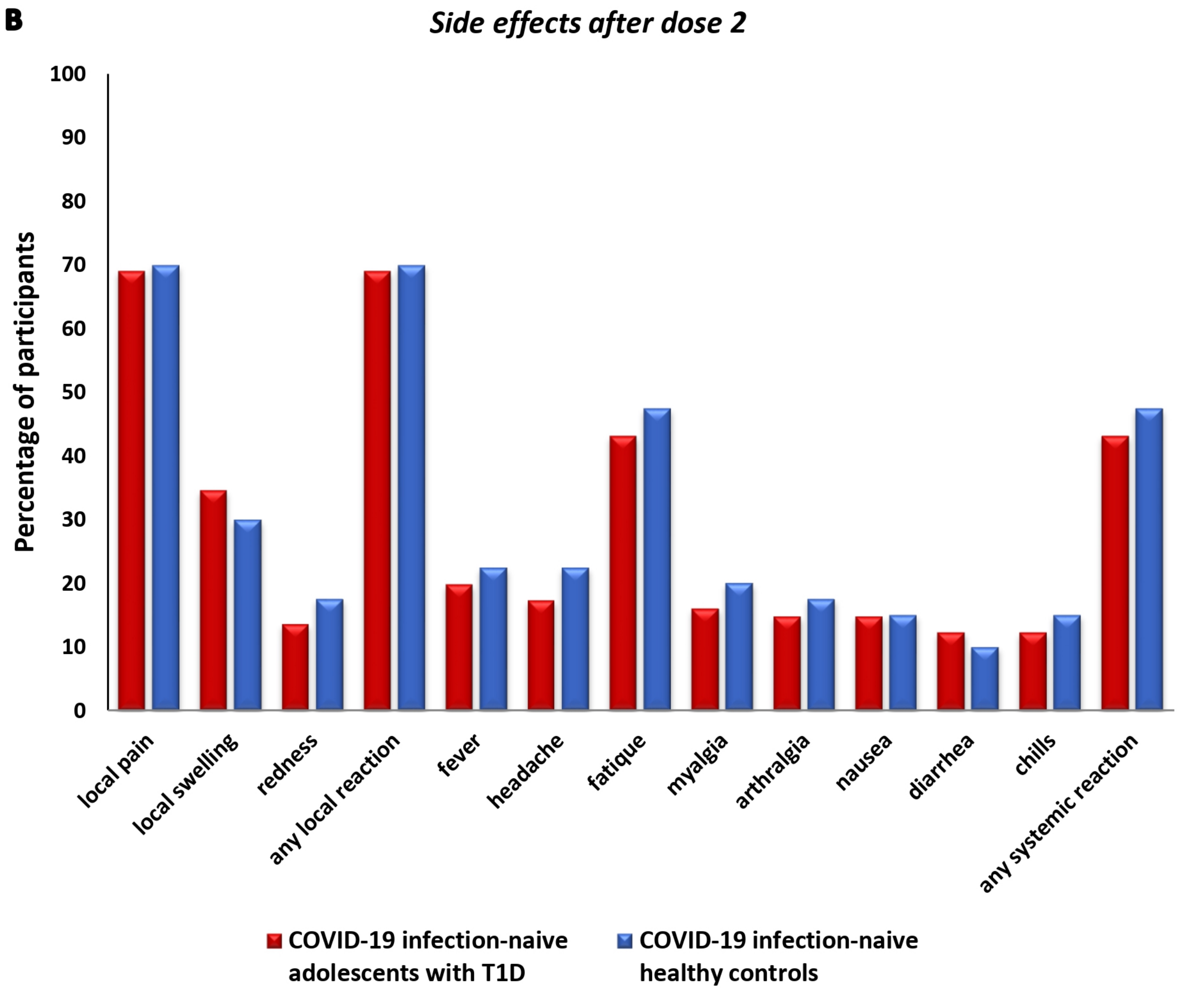

Supplement: Supplementary file 1 [file Image1.pdf]
