# Supplementary figures and images for: Immunogenicity, safety and clinical outcomes of the SARS-CoV-2 BNT162b2 vaccine in adolescents with type 1 diabetes
Source: Front Pediatr. 2023 Jun 26;11:1191706. doi: 10.3389/fped.2023.1191706 (PMC10331611; doi:10.3389/fped.2023.1191706)

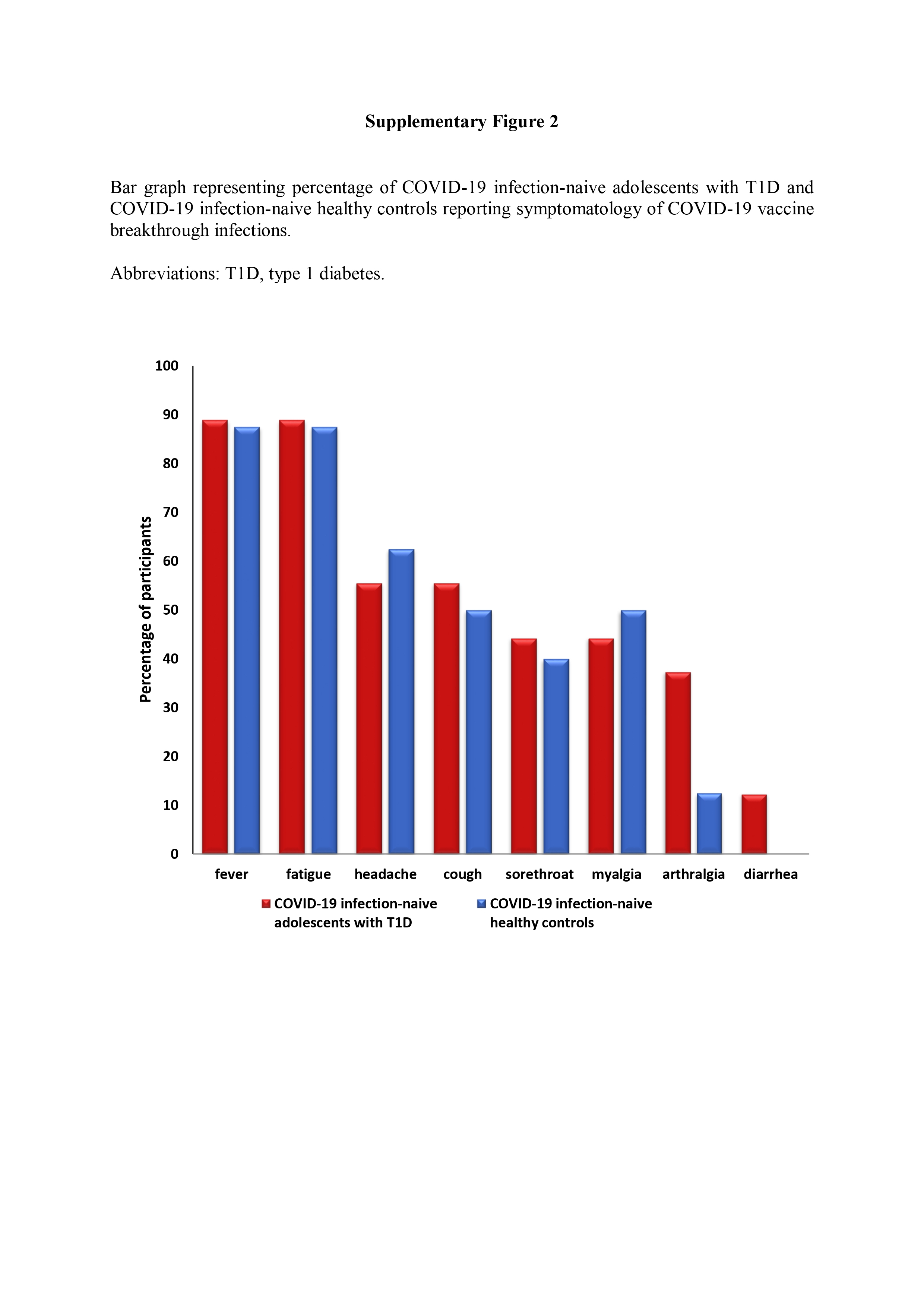

Supplement: Supplementary file 2 [file Image2.jpeg]
